# Optical detection for magnetic field using Ni-subwavelength grating on SiO_2_/thin-film Ag/glass structure

**DOI:** 10.1038/s41598-020-74202-w

**Published:** 2020-11-09

**Authors:** Yuusuke Takashima, Kohei Moriiwa, Masanobu Haraguchi, Yoshiki Naoi

**Affiliations:** 1 Graduate School of Science and Technology, Tokushima University, 2-1 Minami-josanjima, Tokushima, 7708506 Japan; 2 Graduate School of Advanced Technology and Science, Tokushima University, 2-1 Minami-josanjima, Tokushima, 7708506 Japan; 3 Institute of Post-LED Photonics, Tokushima University, 2-1 Minami-josanjima, Tokushima, 7708506 Japan

**Keywords:** Optical sensors, Nanophotonics and plasmonics

## Abstract

An optical sensor for magnetic field detection using Ni-subwavelength grating (SWG) on SiO_2_/Ag-thin-film/glass substrates was experimentally developed on the basis of the re-radiation condition of surface-plasmon-polaritons (SPPs) at Ag surfaces. The fabricated sample showed two dips in the reflection spectra associated with SPP excitation, and the optical response exhibited good agreement with that simulated by the finite-difference time-domain method. The reflectivity at one of the dip wavelengths varied minimally with the application of the magnetic field, whereas that at the other dip wavelength significantly decreased owing to the large electric field overlap of SPP with the magnetized Ni-SWG. As a result, a magnetic field on the order of a few mT could be detected with a simple normal-incidence optical system.

## Introduction

Subwavelength structures, whose sizes are smaller than the incident wavelength, have attracted research interest in many fields as their optical characteristics can be artificially controlled by adjusting the structure geometry. The photonic bands inside subwavelength structures differ considerably from those in free space and largely depend on the structural size, shape, periodicity, and the surrounding material refractive index. Utilizing the photonic bands in subwavelength structures, extraordinary features, such as high reflectivity mirror with broad band^1–5^, polarizer^6–10^, ultrahigh-Q resonator^11–14^, meta-lens^15–18^, and sensing^19–25^, have been realized.

Several groups have reported the enhancement of the magneto-optical (MO) effect using subwavelength structures composed of ferromagnetic materials^26–34^. The MO effect is qualitatively and approximately explained by the Lorentz force. More precisely, quantum physics is necessary to investigate the magnetic characteristics of ferromagnetic materials. The Lorentz force affects the electron polarization induced by the electric field of the light and non-diagonal components of the dielectric tensor appear^30,35^, such as Faraday and Kerr rotations, due to the MO effect. To enhance the MO response, a localized electric field in the subwavelength structure has been frequently employed. Recently, dark mode resonance was utilized in multilayer magnetoplasmonic crystals to enhance the MO effect, and 135-times folder MO response was achieved than that without the resonance^34^.

In our previous works, we experimentally developed a magnetic field sensor utilizing MO enhancement of Ni-subwavelength grating (SWG)^36–38^. The sensor can detect a magnetic field of tens mT order experimentally with simple normal incident geometry. The compactness and simple geometry of our sensor are very useful for portable applications in many fields, including biology, medical science, and engineering. However, the sensitivity and accuracy of magnetic field measurements are still not sufficient for the practical use of magnetic field sensing applications. Thus, further improvement of sensitivity is critical for the applications sensing of magnetic field.

In this work, the improvement of the sensitivity of magnetic field sensor was conducted using optical resonance in Ni-SWG on a SiO_2_/thin-film-Ag/glass structure. To realize the high sensitivity of magnetic field sensing, we designed the structural parameters of the proposed structure using a ω–k dispersion relation of surface plasmons (SPs) at the SiO_2_/Ag and Ag/glass interfaces. We also investigated the electromagnetic field in the structure using the finite-difference time-domain (FDTD) method to understand the optical property of the designed structure. The calculation results indicated that the two reflectivity dips originated from long-range (LR) and short-range (SR) SP mode excitations. The designed structure was fabricated using lithography technique with an electron-beam (EB), and the two dips in the reflection spectrum were experimentally obtained at the wavelengths of 502 (LRSP) and 570 nm (SRSP). The reflectivity of the dip at the wavelength of 502 nm considerably varied with the applied external magnetic field during ten measurements, and our sensor detected several mT of magnetic field using simple optical setups.

## Results

### Operating principle

**Figure 1:**
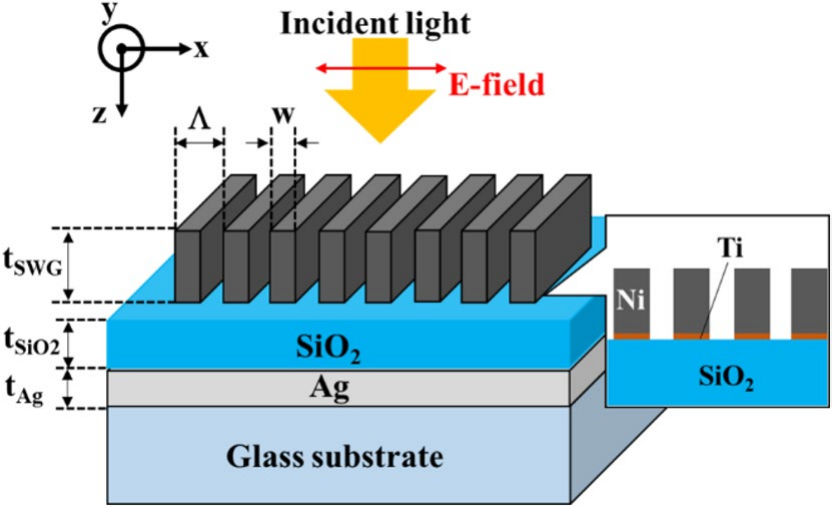
The geometry of the proposed sensor.

The structural parameters of the Ni-SWG and SiO_2_/Ag/glass structure were designed for magnetic field sensing. Figure 1 illustrates the geometry of our sensor. SiO_2_ and Ag films were deposited on the glass substrate. Ni was selected as the ferromagnetic-SWG material because of its large saturation magnetization^39^. We arranged the Ni-SWG on the top of the SiO_2_/Ag/glass multi-layers. A 5 nm Ti film was also inserted between the Ni-SWG and the SiO_2_ film to connect both firmly. The symbols Λ, w, t_SWG_, t_SiO2_, t_Ag_, represent the grating period, grating finger width, grating height, and thickness of SiO_2_ film and Ag film, respectively.

SP, the surface modes composed of collective electron oscillation, exists at the interfaces of SiO_2_/Ag and Ag/glass substrates. For the structure, a p-polarized incident light entered vertically. The electric field of p-polarized light was vertical to the periodic fingers of the SWG, as shown in Fig. 1. The SWG modulated the lateral wavenumber (namely, x-direction in Fig. 1) of the incident light, and several order diffractions occurred. In particular, all higher-order diffractions except for 0th-orders (namely, transmission and reflection) had an evanescent form owing to the shorter SWG period than that of the incident wavelength. When the lateral wavenumber of the diffraction of the higher-order coincided with that of SP, the diffraction coupled with the SP and formed surface-plasmon-polariton (SPP). The excitation of SPP led to decreasing reflectivity of the proposed structure because the incident light energy transformed into SPPs. As a result, a dip in the reflection spectrum of the structure appeared.

The excited SPP propagated along the surface of the Ag film. The SPP was re-radiated by the SWG during the propagation along the Ag surface. The interference between 0th diffraction (reflection) and re-radiated waves exhibited a considerable influence on the reflection spectrum of the structure. The excitation and re-radiation conditions of the SPP are sensitive for the polarization state of the light. As we applied the external magnetic field to the structure, the polarized direction of the light rotated because of the non-diagonal dielectric tensor of the magnetized Ni-SWG. Thus, the reflected intensity at the reflection dip originated from the SPP excitation significantly varies for the applied magnetic field.

In particular, the SPs at SiO_2_/Ag and the Ag/glass interacted and coupled, considering that the Ag film was thinner than the penetration depth of each mode. As a result, LRSP and SRSP were excited^40,41^. Moreover, the electric field of LRSP mostly seeped toward the dielectric material side (SiO_2_ and glass substrate in this study), whereas the dominant field of the SRSP was concentrated in the Ag layer. Thus, we expected that the LRSP mode was particularly influenced by the magnetization of Ni because its electric field largely overlapped with that of the Ni-SWG.

### Design of SiO_2_/thin-film-Ag/glass structure for magnetic field sensing

To excite the LRSP in the visible-wavelength region, we determined the geometrical parameters of our structure using the ω–k dispersion relation of SP at the SiO_2_/Ag/grass substrate. The detail of the dispersion relations was discussed in previous publication^40,41^. The visible light was employed as incident light owing to its ease of treatment. The information on the dielectric functions of Ni, Ag and SiO_2_ were found from literatures^42,43^. The glass was assumed to commercialized glass (D263 T eco Thin Glass: SCHOTT). The structural parameters were set to grating period Λ = 300 nm, height t_SWG_ = 100 nm, and width w = 150 nm, and thicknesses of t_Ag_ = 30 nm and t_SiO2_, = 70 nm, respectively.

**Figure 2:**
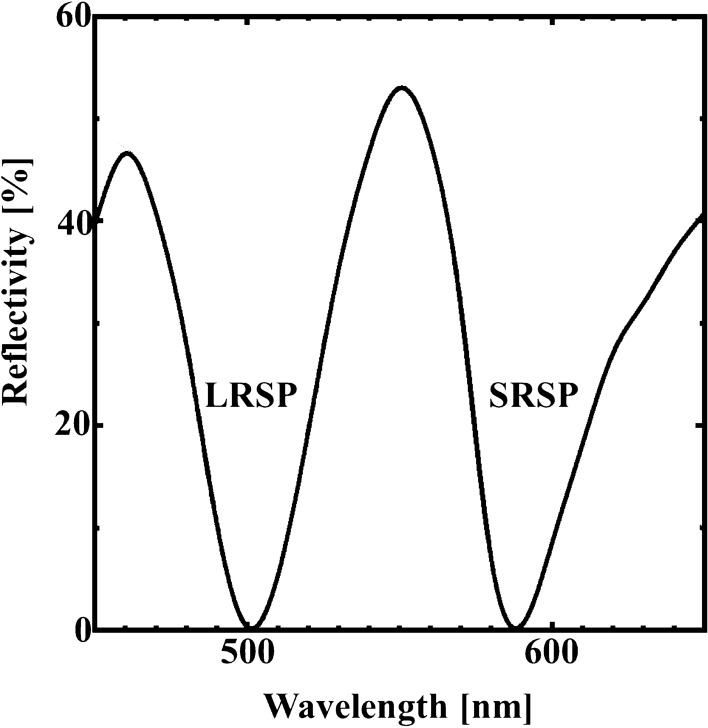
Normal reflection spectrum of the designed sensor using numerical calculation based on FDTD method. Poynting vector was used to evaluate the reflectivity.

**Figure 3:**
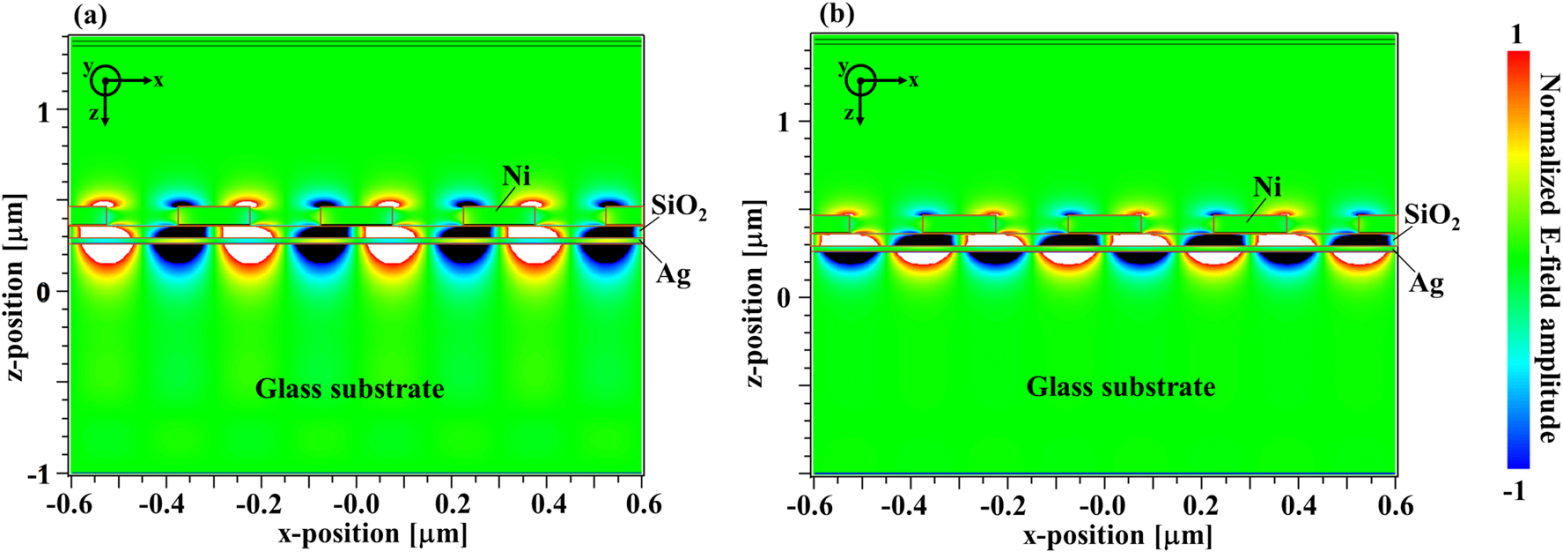
The z-component electric field distribution (**a**) at the wavelength of 500 nm and (**b**) at the wavelength of 590 nm. The amplitude of field is normalized by the incident field amplitude.

Figure 2 shows the calculated reflection spectrum of our designed structure using FDTD method. In the calculation, the propagation direction of the p-polarized plane wave was the + z-direction and entered the designed structure normally. The calculation detail is described in method section. In Fig. 2, we found two deep reflection dips at the wavelength of 500 nm and 590 nm, respectively. The reflected intensities vanished at these wavelengths. We also illustrate the z-component electric field distributions at the wavelengths of 500 nm (LRSP) and 590 nm (SRSP), as shown in Fig. 3a,b, respectively.

The z-components of the electric field appeared at Ni-SWG and Ag surfaces despite the incident light propagating along the z-direction. The electric field colored by black and white mean the saturated field. The field patterns indicated that the dips found at the wavelength of 500 nm and 590 nm resulted from LRSP and SRSP, respectively^40,41^. We also found that the electric field concentrated considerably on the SiO_2_/Ag and Ag/glass surfaces. The distribution indicated that the diffracted lights coupled with the SPPs, which propagated along the surface of Ag. In particular, the most of electric field of the LRSP largely concentrated into the Ni-SWG structure, while that of SRSP spread to air-gap region of the SWG (See the field in the SiO_2_ layer in Fig. 3a,b).

This considerable overlap of the electric field implied that the re-radiation conditions of LRSP were significantly affected by the magnetization of Ni-SWG.

Moreover, we calculated the effect of the Ni magnetization on the electromagnetic field distributions around our sample at two dip wavelengths (500 nm and 590 nm). When magnetic field was applied to our sample (magnetic field and light propagation direction are z-direction, as shown in method section), Ni dielectric tensor ε_Ni_ is given as following equation.

1 $${\varepsilon }_{Ni}=\left(\begin{array}{ccc}{\epsilon }_{xx}& {\epsilon }_{xy}& 0\\ -{\epsilon }_{yx}& {\epsilon }_{yy}& 0\\ 0& 0& {\epsilon }_{zz}\end{array}\right)$$

where the ε_xx_, ε_yy_, and ε_zz_ are diagonal components and the ε_xy_ and ε_yx_ are non-diagonal components. The relations between these components of dielectric tensor are ε_xx_ = ε_yy_ = ε_zz_ and ε_xy_ = − ε_yx_, respectively. The non-diagonal components ε_xy_ and ε_yx_ are originated via the magnetization of Ni, and these components induces the polarization rotation of the reflected light (Kerr MO effect). As a result, the y-component of the electric field E_y_ is generated when magnetic field was applied to our sample (the incident light originally has only an electric field of x-component, as described in method section). The diagonal and non-diagonal component values are taken from the experimental results in these literatures^42,44^, and ε_xy_ are set to 0.016–0.03i at both wavelengths of 500 nm and 590 nm. Although these values of the Ni dielectric tensor are that of bulk Ni and deviate from that of nano sized Ni, it is enough for the qualitative investigation of the tendency of the light behavior for the Ni magnetization.

**Figure 4:**
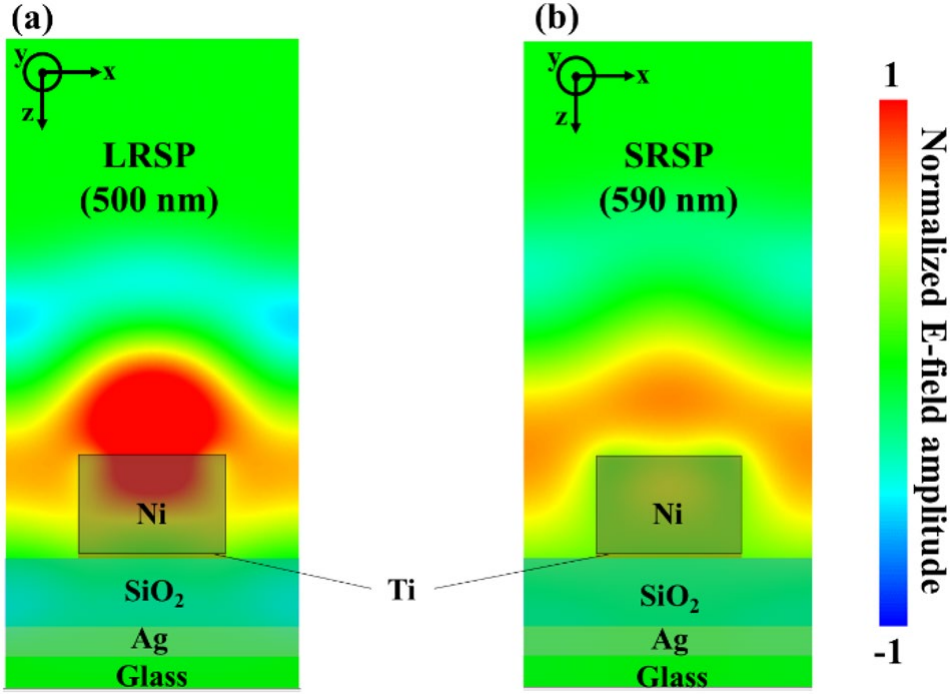
The y-component electric field distribution (**a**) at the wavelength of 500 nm and (**b**) at the wavelength 590 nm. The amplitude of field is normalized by that of LRSP.

Figure 4a,b show the normalized E_y_ distributions our sample at the reflection dips, respectively. As shown in Fig. 4a,b, the E_y_ components appear around our structure at both wavelengths. The distributions indicate that the polarization of the light is rotated by the non-diagonal components of the magnetized Ni-SWG. Especially, the larger amplitude of E_y_ appears at the wavelength of 500 nm (LSPS dip wavelength), and the reflected power of E_y_ component is about 2 times greater than that at the wavelength of 590 nm (SRSP dip wavelength). This result is evidence that the large overlap of the LRSP electric field with Ni-SWG enhances the interaction between magnetization and light, and the re-radiation condition of LRSP is more sensitive than that of SRSP. Thus, we can assume that the reflected intensity at dip resulting from LRSP excitation significantly varies with the applied magnetic field.

### Optical characteristics

**Figure 5:**
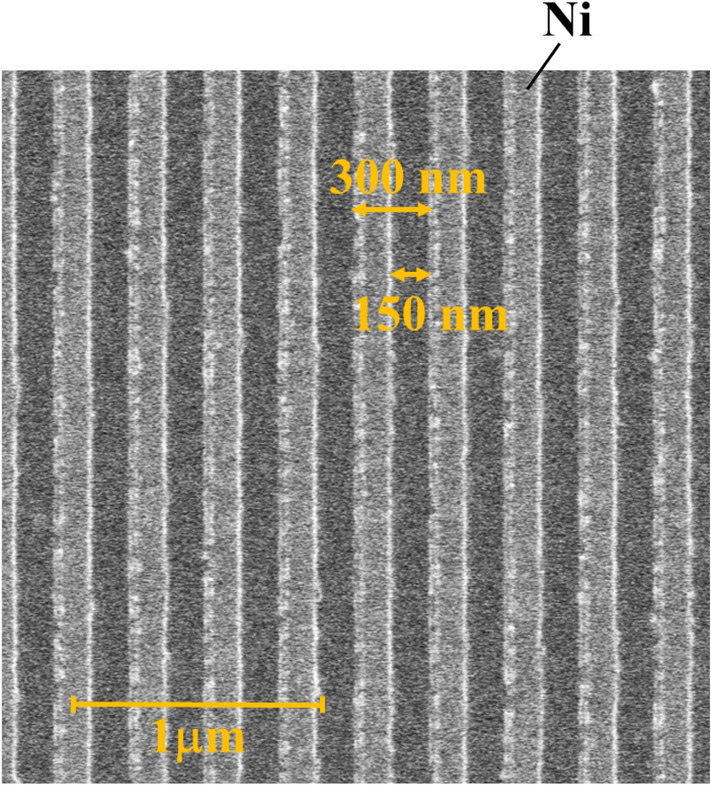
SEM image of surface view of the fabricated sample. The scale bar indicates 1 µm.

We employed traditional lithography techniques with EB for the fabrication of the designed Ni-SWG/SiO_2_/Ag structure. Figure 5 shows the scanned EB microscope (SEM) image of the surface view of the fabricated sample; it also illustrates a 300 nm period and 150 nm line width, respectively.

**Figure 6:**
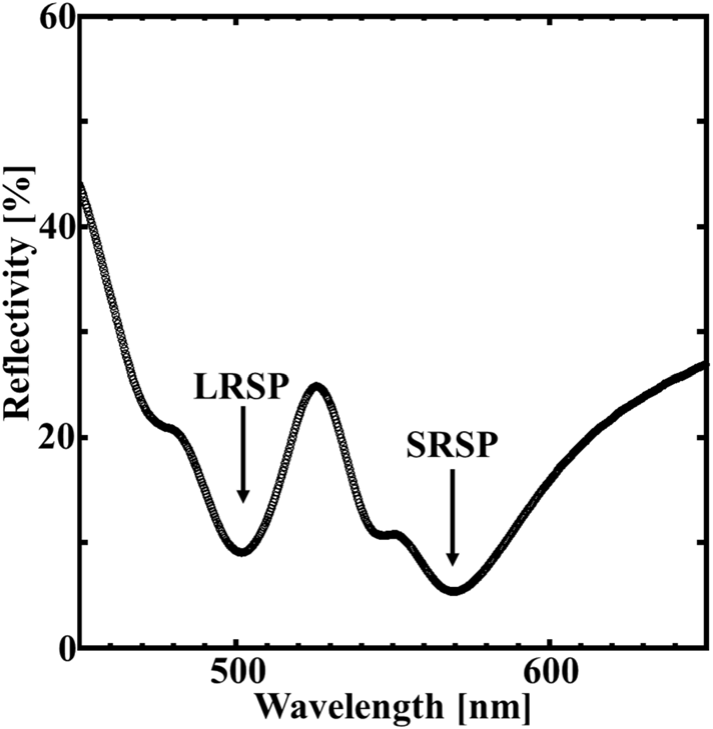
Reflectivity spectrum of the fabricated sample. The reflectivity was evaluated by the Al mirror.

The measured reflection spectrum is shown in Fig. 6. The reflectivity was measured utilizing very simple system with normal incidence. The detail of system is described in method section. The reflectivity value was determined on the basis of the Al mirror (TFA-50C08-4: Sigma). As shown in Fig. 6, the reflectivity of the sample decreased at wavelengths of 502 and 570 nm, and two reflection dips appeared. These results agree well with FDTD calculation results, and the dips at the wavelengths of 502 and 570 nm correspond to LRSP and SRSP excitation, respectively.

### Magnetic field sensing

**Figure 7:**
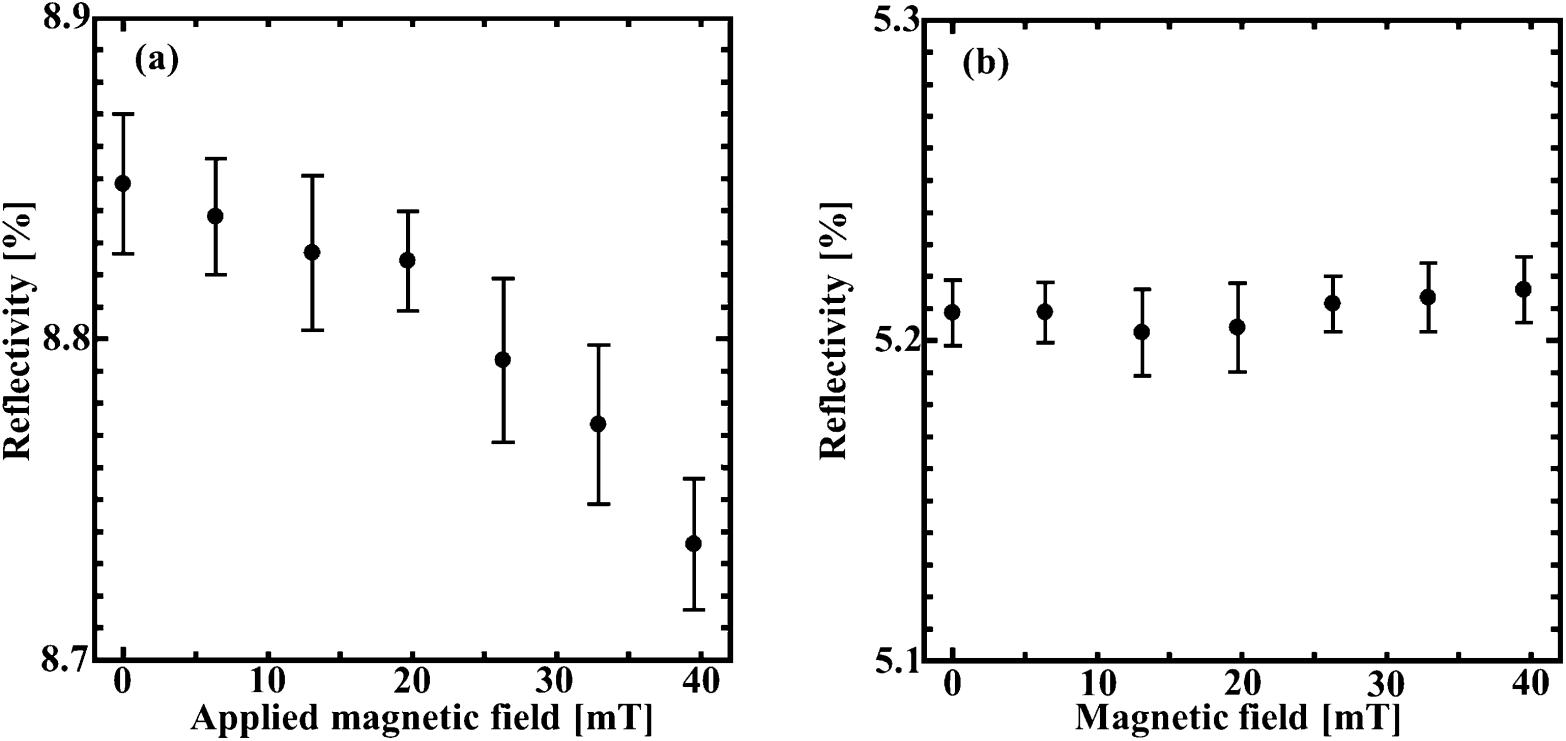
Magnetic field dependence of the reflectivity at (**a**) the wavelength of 502 nm (**b**) 570 nm. The error bars show standard deviation during 10 measurements.

To clarify the magnetic response of the fabricated device, we applied a magnetic field perpendicular to the sample. Figure 7a,b show the magnetic field dependence of the reflectivity at the wavelengths of 502 nm (LRSP) and 570 nm (SRSP), respectively. Magnetic field values of 6.4, 13.1, 19.7, 26.3, 32.9, and 39.5 mT were applied. The filled circles and the error bars indicated the average reflectivity and ± standard deviations during 10 measurements. Figure 7a indicates that the reflectivity at the dip resulting from the excitation of LRSP in our structure decreased with an increase of the value of the applied magnetic field. On the other hand, the reflectivity at the excitation of SRSP depends minimally on the magnetic field up to 39.5 mT, as shown in Fig. 7b. According to the experimental results, we find that LRSP is more sensitive to the magnetic field rather than SRSP and that the designed sensor with a very simple optical setup can distinguish magnetic fields of several mT. This sensitivity performance of our sensor is almost equality high to other optical sensors despite its simple and compact measurement system^45–47^.

## Discussion

The high sensitivity of our sensor can be qualitatively explained by considering the enhanced Lorentz force because of the electric field of LRSP. The polarization direction of the electrons was influenced by the Lorentz force caused by the magnetization of Ni-SWG, and non-diagonal tensor of the dielectric constant were generated. The significant overlap of LRSP electric field with Ni-SWG significantly contributed to the enhancement of the non-diagonal dielectric tensor, because the Lorentz force increased with an increase in the electric field. As a result, the excitation and re-radiation condition of LRSP sensitively varied by the applied magnetic field for Ni-SWG. The high sensitivity and simplicity of our sensor are suitable for the practical use of the magnetic field sensor, and our sensor open new integration device concepts for magnetic field detection.

## Conclusion

In conclusion, we experimentally developed a highly sensitive magnetic field sensor incorporating Ni-SWG/SiO_2_/Ag structure. The sensor was designed based on the ω–k relation of SPP modes at Ag surfaces to excite the modes in visible-wavelength regions. The numerically calculated reflection spectrum of the designed structure indicated the two reflectivity dips caused by LRSP and SRSP; in addition, the electric field distribution of LRSP largely overlapped with that of the Ni-SWG. The calculated electric field distribution also predicted the larger MO response of LRSP than that of SRSP. We fabricated the designed Ni-SWG on the SiO_2_/Ag/glass substrate using the EB lithography technique and obtained experimental reflectivity dip values at wavelengths of 502 (LRSP) nm and 570 nm (SRSP), respectively. The reflectivity at the LRSP dip dramatically decreased as the value of applied magnetic field for the sample increased, and several mT of the magnetic field were detected using simple optical setups. Moreover, these results indicate that the magnetically modulation depth of the reflection spectrum can be improved by adjusting the SiO_2_ thickness between Ag film and Ni-SWG because the overlap of LRSP electric field with the SWG strongly depend on the thickness of SiO_2_ spacer. In the further work, we will optimize the SiO_2_ thickness for higher sensitivity of magnetic field sensing and will report the optimization elsewhere.

## Methods

### FDTD calculation

**Figure 8:**
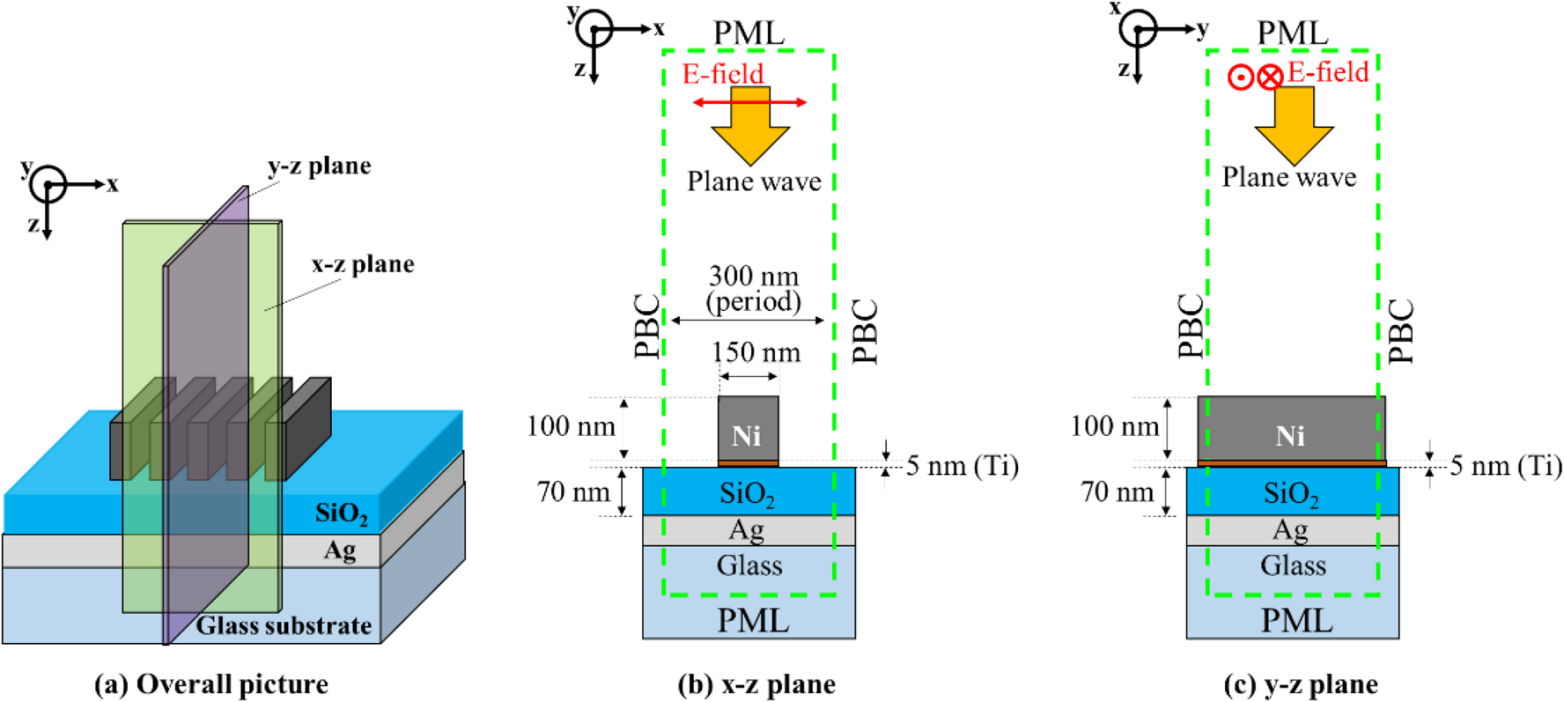
Schematic diagram of FDTD simulation model (**a**) overall picture, (**b**) x–z plane (**c**) y–z plane.

We investigated the reflection characteristics of the designed structure using FDTD numerical simulation (Fullwave: R-Soft and Poyinting for Optics: FUJITSU) for electromagnetic field distribution and the interaction between magnetization of the Ni-SWG and the light. Figure 8 shows the model for the FDTD simulations. The area surrounded by green dashed lines represents the calculation region, and the dashed lines mean boundaries in the simulations. We postulated that the structural length is infinite for y-direction and the structure repeats for x-direction in the simulation. Hence, we employed periodic boundary conditions (PBC), in which the electromagnetic fields infinitely repeat, as x- and y-boundaries. The thickness of the glass substrate was also assumed infinite thick, and perfect matched layer (PML) boundary conditions, in which the electromagnetic field was perfectly absorbed, were used for z-boundaries. These assumptions were justified because the actual lengths of structure for x- and y-directions were much larger than the incident wavelength. In the simulations, spatial mesh size Δx, Δy, Δz were 2 nm, and time step Δt was set to 0.4688 × 10^−13^ s for the convergence of the solution (time step Δt was set to 0.0035 × 10^−15^ s for investigation of the magnetic response). The incident plane wave was polarized along x-direction. The propagation direction of the incident wave was the + z-direction and entered the designed structure normally. The Poynting vector was utilizing for the estimation of the reflected light intensity.

### Fabrication

We fabricated the Ni-SWG and SiO_2_/Ag/glass structures. First, Ag film with 30 nm-thickness was thermally evaporated on the glass substrate (D263 T eco Thin Glass: SCHOTT). The 70 nm-thickness SiO_2_ film was deposited on the Ag film using EB evaporation technique. Second, the EB lithography resist film (ZEP520A: Zeon) was spin-coated on the SiO_2_ film at 3000 rpm for 90 s. The SWG pattern was drawn by the resist film EB lithography techniques with an acceleration voltage of 50 kV. The area size of the SWG pattern was a square region of 300 µm × 300 µm. Subsequently, we formed the SWG resist pattern using a developer (ZED-N50: Zeon) with 20 °C. Finally, Ti and Ni films with thicknesses of 5 and 100 nm were evaporated on the patterned resist film, respectively, and the resist film was removed using an *N*-methyl-pyrrolidone solution.

### Optical and magnetic characterization

**Figure 9:**
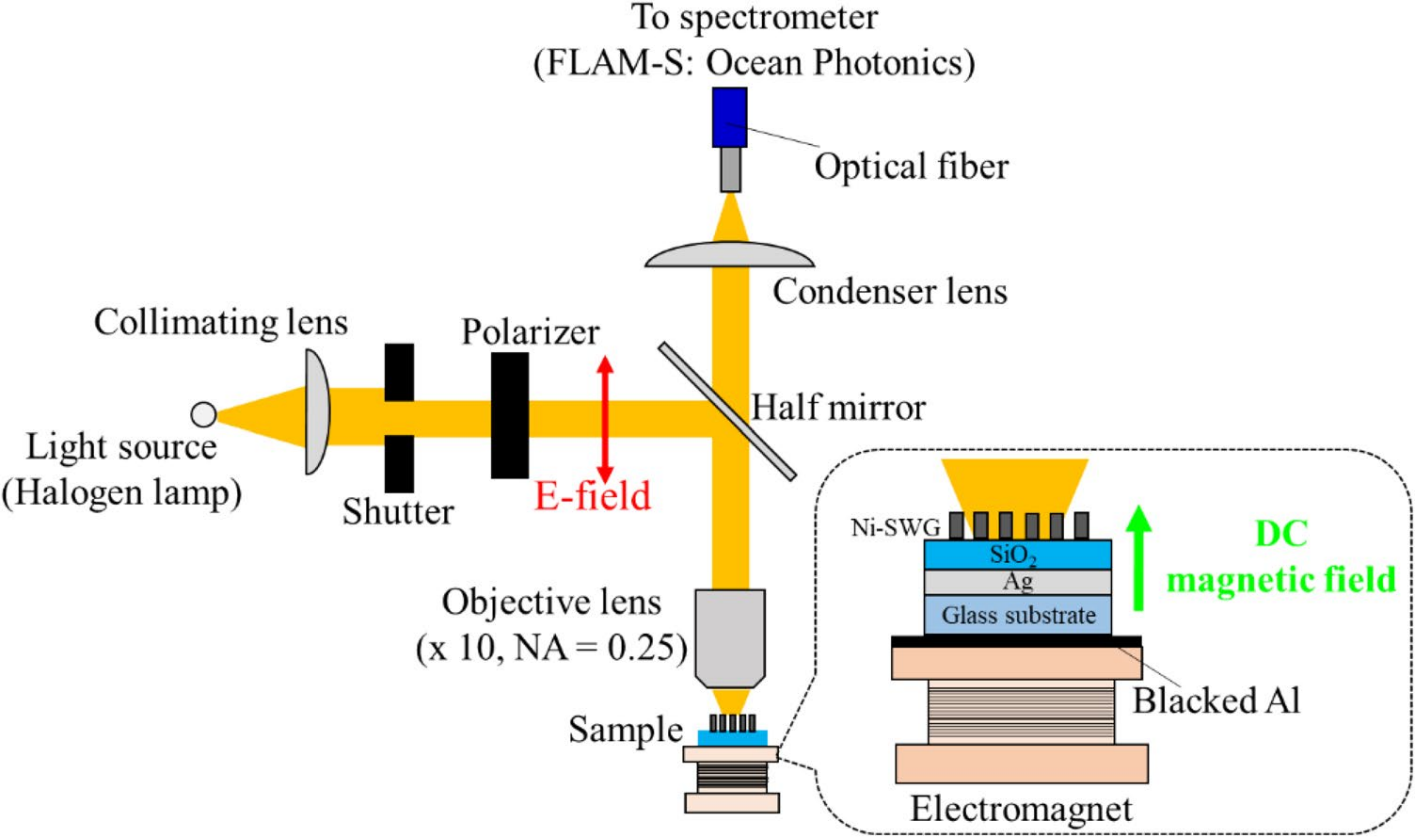
Measurement system for reflection spectrum and magnetic response.

We investigated the reflection spectrum and magnetic response of the fabricated sample for normal incident light. The optical irradiation system is shown in Fig. 9. To apply the magnetic field, we set the sample at top of the electromagnet with the iron core.

The insertion of blacked Al film between the sample and the electromagnet prevented reflection at the surface of the magnet. A halogen lamp was employed as the visible light source. The light from the lamp passes through the shutter in order to irradiate the light into only SWG region. The light was p-polarized by polarizer, and the p-polarized incident light was irradiated on the fabricated pattern area by an objective lens (magnification: × 10, numerical aperture (NA): 0.25). The light reflected at the sample entered the spectrometer (FLAM-S: Ocean Photonics). The magnetic field perpendicular to the sample (the field direction is from glass substrate to structure) was applied by flowing a current to the electromagnet.
